# Low-frequency repetitive transcranial magnetic stimulation for children and adolescents with first-episode and drug-naïve major depressive disorder: A systematic review

**DOI:** 10.3389/fpsyt.2023.1111754

**Published:** 2023-02-08

**Authors:** Wei Zheng, Xian-Jun Lan, Zhen-Juan Qin, Xin-Hu Yang, Zhan-Ming Shi

**Affiliations:** ^1^The Affiliated Brain Hospital of Guangzhou Medical University, Guangzhou, China; ^2^The Brain Hospital of Guangxi Zhuang Autonomous Region, LiuZhou, China; ^3^Chongqing Jiangbei Mental Health Center, Chongqing, China

**Keywords:** rTMS, major depressive disorder, first episode, children, adolescents

## Abstract

**Objective:**

This systematic review of randomized controlled trials (RCTs) was conducted to explore the therapeutic effects and safety of active low-frequency repetitive transcranial magnetic stimulation (LF-rTMS) versus sham LF-rTMS in children and adolescent patients with first-episode and drug-naïve (FEDN) major depressive disorder (MDD).

**Methods:**

A systematic literature search was performed, and data were extracted by two independent researchers. The coprimary outcomes were study-defined response and remission.

**Results:**

A systematic search of the literature yielded 442 references, of which 3 RCTs (130 children and adolescents with FEDN MDD, 50.8% male, and mean age range from 14.5 to 17.5 years) met the inclusion criteria. Among the two RCTs (66.7%, 2/3) examining the effects of LF-rTMS on study-defined response and remission and cognitive function, active LF-rTMS was more efficacious than sham LF-rTMS in terms of study-defined response rate and cognitive function (all *p* < 0.05) but not regarding study-defined remission rate (all *p* > 0.05). No significant group differences were found with regard to adverse reactions. None of the included RCTs reported the dropout rate.

**Conclusion:**

These findings preliminarily found that LF-rTMS could benefit children and adolescents with FEDN MDD in a relatively safe manner, although further studies are warranted.

## Introduction

Major depressive disorder (MDD), as a leading cause of global disease burden (1), affects approximately 5–15% of children and adolescents (2). Children and adolescents with MDD are usually related to school dropout, pregnancy/parenthood, and unemployment (3). Furthermore, individuals suffering from MDD in childhood and adolescence have a relatively high risk for chronic recurrence, suicide, and long-term psychosocial impairment in adulthood (2, 4–6). Therefore, improvements in treating MDD among children and adolescents should positively affect public health.

Initial treatment of children and adolescents with MDD may include a selective serotonin reuptake inhibitor (SSRI) or cognitive-behavioural therapy (CBT) (7). A Treatment for Adolescents with Depression Study (TADS) randomized controlled trial (RCT) found that CBT combined with fluoxetine provided a more favorable tradeoff between risk and benefit in adolescent patients with MDD than either treatment alone (7). However, up to 40% of adolescents suffering from MDD fail to respond to traditional treatment (8, 9). As a result, new and effective treatment approaches for MDD patients among children and adolescents are urgently needed.

Repetitive transcranial magnetic stimulation (rTMS), as a noninvasive brain stimulation, is gaining attention in treating adults suffering from various conditions, including MDD and obsessive–compulsive disorder (OCD) (10–12). Repetitive transcranial magnetic stimulation uses a magnetic field to stimulate the cortex and depression-related areas with electrical currents and alter dysfunctional brain patterns (13, 14). Numerous RCTs have demonstrated the therapeutic effects of rTMS in adult patients with treatment-refractory depression (TRD) (15, 16). The utility of rTMS for adult patients with MDD and OCD who did not respond to medications has been approved by the US FDA (9, 17). Accumulating evidences found that rTMS also could accelerate the rapidity of the antidepressant response in adult patients suffering from first-episode MDD (18, 19). Case reports/series (20–22) and observational studies (23–28) reported that rTMS appeared to be suitable for children and adolescents diagnosed with MDD. However, the findings of RCTs (29–31) examining the therapeutic effects and safety of active low-frequency rTMS (LF-rTMS) versus sham LF-rTMS for children and adolescents with first-episode and drug-naïve (FEDN) MDD have been inconsistent.

Therefore, the primary aim in this systematic review of RCTs was to investigate the therapeutic effects and safety of active LF-rTMS versus sham LF-rTMS for children and adolescents with FEDN MDD. We hypothesized that active LF-rTMS would be more efficacious than sham LF-rTMS in ameliorating depressive symptoms in FEDN MDD patients among children and adolescents.

## Methods

### Search strategy and selection criteria

To identify studies for inclusion in this systematic review, two researchers (ZJQ and XJL) independently searched Chinese Journal Net, WanFang databases, PsycINFO, Cochrane Library, PubMed, and EMBASE through November 4 2022. The search terms are listed in Appendix S1. Additionally, we manually searched reference lists of previous reviews (2, 9, 32) and the included RCTs (29–31) on active LF-rTMS versus sham LF-rTMS for children and adolescent patients with FEDN MDD.

In accordance with PRISMA (Preferred Reporting Items for Systematic Reviews and Meta Analyses) guidelines (33), we included studies that fulfilled the following *PICOS* criteria. *P*articipants: Children (6–11 years) (34) and adolescents (12–25 years) (35) with a diagnosis of first-episode MDD who did not receive any antidepressant treatment. In line with the methodology of a recent systematic review (35), adolescents were defined as those who are 12–25 years old rather than 13–18 years old. ***I***ntervention versus ***C***omparison: active LF-rTMS versus sham LF-rTMS. Outcomes: The coprimary outcomes were study-defined response (i.e., at least 50% reduction in Hamilton Depression Rating Scale (HAMD) scores) and remission (i.e., at least 75% reduction in HAMD scores). Additional outcomes were cognitive function, dropout rate, and adverse events. ***S***tudy: Only published RCTs on active LF-rTMS versus sham LF-rTMS for children and adolescents (6–25 years) with FEDN MDD were eligible for inclusion. Studies focusing on active LF-rTMS versus antidepressants (36) or high-frequency rTMS (HF-rTMS) combined with antidepressants versus antidepressant monotherapy (14) were excluded. Review articles and case reports/series were also excluded.

### Data extraction

Two independent researchers (ZJQ and XJL) performed the data extraction from each included RCT, and any disagreements were resolved by joint discussion. We extracted data using a standardized form including author, year of publication, study design, rTMS protocol, and primary and secondary outcomes. Additional data were requested by contacting the original study author(s), if necessary.

### Study quality assessment

The quality of the RCTs was independently assessed by the same two researchers (ZJQ and XJL) using the Jadad scale (37) and the Cochrane risk of bias (38). As reported previously (39), RCTs were considered “high quality” when the Jadad score was ≥3.

## Results

### Study selection

**Figure 1:**
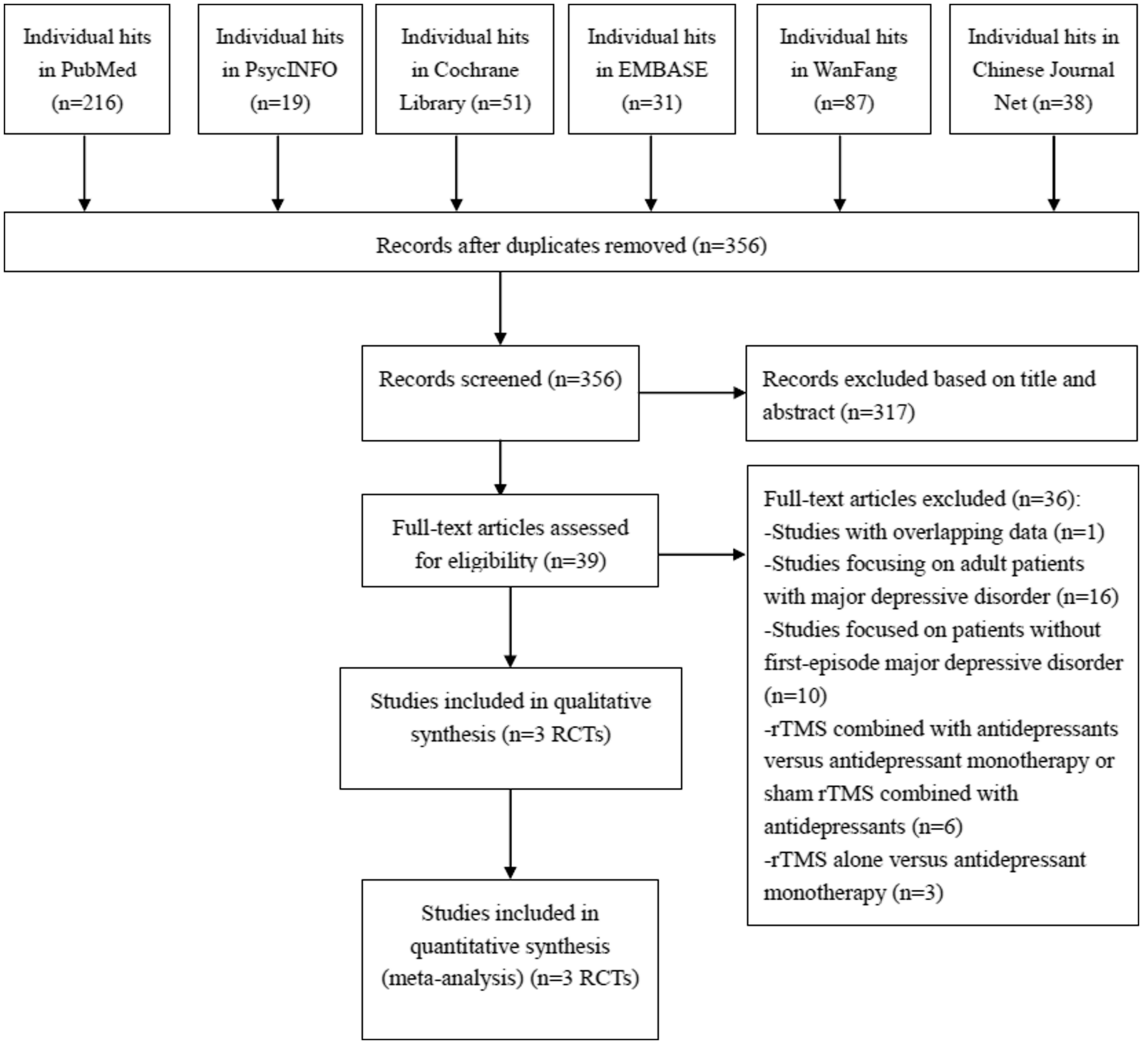
PRISMA flow diagram.

Our initial search of the above English and Chinese databases retrieved 442 references (Figure 1). Finally, 3 RCTs (29–31) conducted in China met the inclusion criteria of this systematic review. The screening process for the literature is presented in Figure 1.

### Study characteristics

The participant characteristics and LF-rTMS parameters of the three included RCTs (29–31) are summarized in Table 1. The studies (*n* = 130) were conducted between 2015 and 2019, comparing active LF-rTMS (*n* = 65) and sham LF-rTMS (*n* = 65) for children and adolescents with FEDN MDD. Their mean ages ranged from 14.5 to 17.5 years, and more than half (50.7%) of the children and adolescents with FEDN MDD were male. The LF-rTMS treatment duration varied from 10 days (2 RCTs (29, 30)) to 20 days (1 RCT (31)). The detailed LF-rTMS protocol of each included RCT is summarized in Table 1.

**Table 1:** Participant characteristics and low-frequency repetitive transcranial magnetic stimulation (LF-rTMS) parameters of each study included in this systematic review.

Study (country)	Number of patients^a^	Participants: -Diagnosis -Diagnostic criteria -Setting	-Illness duration (months) -Male (%)	Mean age (years) (range)	LF-rTMS treatment duration (days)	Intervention versus control groups; number of patients (*n*)	-Intensity (%MT) -Frequency (Hz)	-Site	-Number of trains per day -Train duration (s) -Intertrain duration (s)	-Pulses per session -Number of sessions -Total pulses	Jadad score
(29) (China)	40	-FEDN MDD -DSM-IV -NR	−1.1 −45	14.8 (12–18)	10	1. Active LF-rTMS; *n* = 20 2. Sham stimulation; *n* = 20	−70 −0.5	R-DLPFC	−40 −10 −2	−200 −10 −2000	1
(31) (China)	60	-FEDN MDD -CCMD-3 -NR	-NR −55	14.5 (10–16)	20	1. Active LF-rTMS; *n* = 30 2. Sham stimulation; *n* = 30	−100 −1	R-PFC	−80 −10 −5	−800 −20 −16,000	1
(20) (China)	30	-FEDN MDD -DSM-IV -NR	−6.3 −50	17.5 (15–21)	10	1. Active LF-rTMS; *n* = 15 2. Sham stimulation; *n* = 15	−80 −1	R-DLPFC	−15 −40 −20	−600 −10 −6,000	2

a Overall number of participants.

CCMD-3 = Chinese Classification and Diagnostic Criteria of Mental Disorders 3rd edition; DSM-IV=Diagnostic and Statistical Manual of Mental Disorders 4th edition; FEDN = first-episode and drug-naïve; MDD = major depressive disorder; MT = motor threshold; NR = not reported; R-DLPFC = right dorsolateral prefrontal cortex; R-PFC = right prefrontal cortex; LF-rTMS = low-frequency repetitive transcranial magnetic stimulation.

### Assessment of study quality

**Figure 2:**
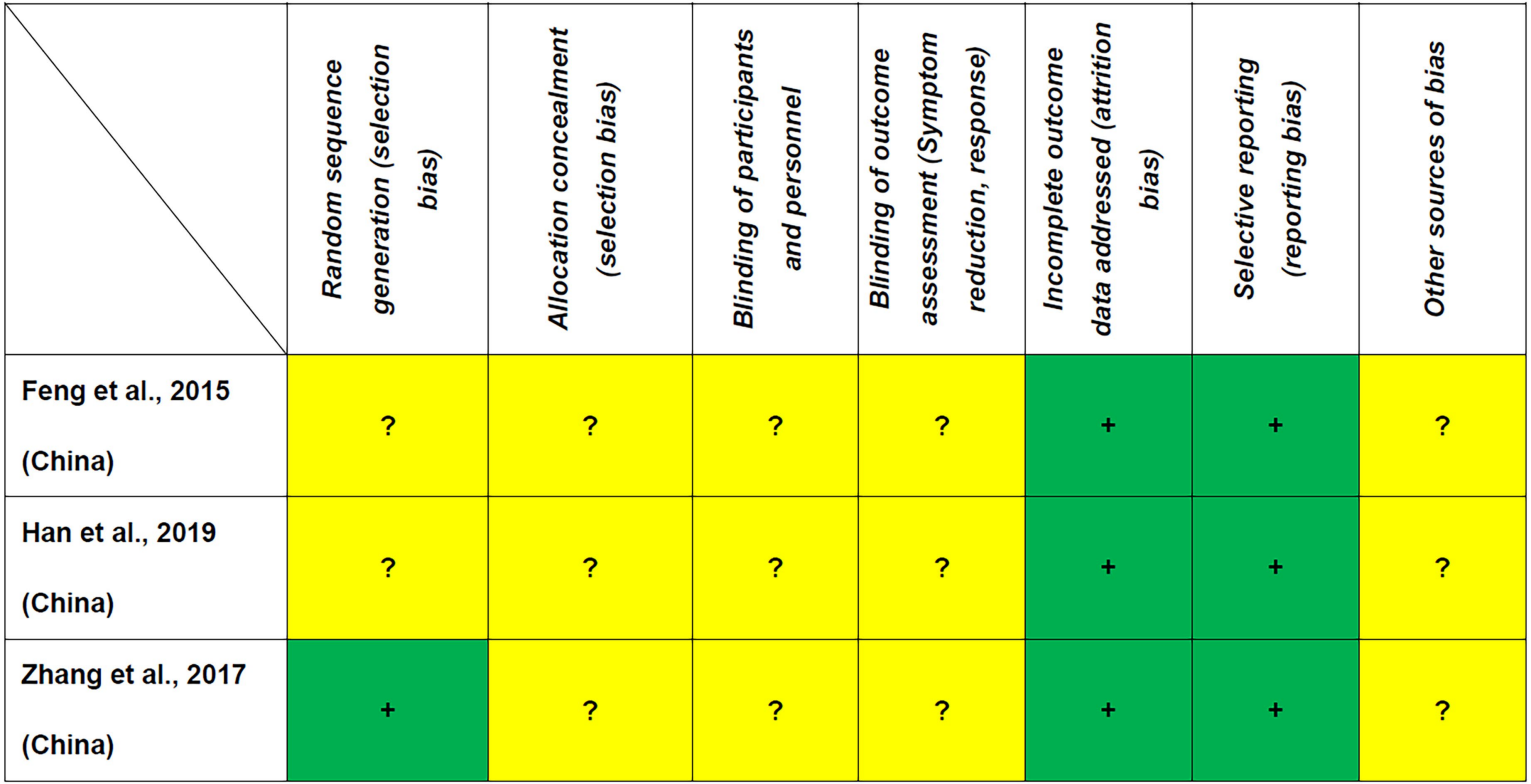
Cochrane risk of bias +: Low risk of bias, −: High risk of bias,?: Unclear risk of bias, nd: not determined.

As shown in Table 1, the Jadad score ranged from 1 (2 RCTs (29, 31)) to 2 (1 RCT (30)); thus, none of the included RCTs fulfilled the criteria of high quality. All RCTs were rated as low risk regarding attrition and reporting bias according to the Cochrane risk of bias (Figure 2).

### Study-defined response and remission

Among the included three RCTs, one RCT (33.3%, 1/3) (29) did not report the rates of study-defined response and remission (Table 2) and found a significant superiority of active LF-rTMS over sham LF-rTMS in improving the depression subfactor scores of the Children’s depression inventory (CDI; all *p* < 0.05). In Han et al.’s study (31), a significant superiority of active LF-rTMS over sham LF-rTMS was found for study-defined response (40.0 vs. 13.3%; *p* < 0.05) between active LF-rTMS and sham LF-rTMS but not for study-defined remission (13.3 vs. 3.3%; *p* > 0.05). Similarly, a significant superiority of active LF-rTMS over sham LF-rTMS was found for study-defined response (46.7 vs. 0%, *p* < 0.05) but not for study-defined remission (0 vs. 0%, *p* > 0.05) in Zhang et al.’s study (30).

**Table 2:** Active versus sham LF-rTMS for adolescent patients with FEDN depression: study-defined response and remission.

Study	Treatment outcomes	Defination	Active LF-rTMS group	Sham stimulation group	Findings
(29) (China)	Study-defined response	NR	NR	NR	NA
	Study-defined remission	NR	NR	NR	NA
(31) (China)	Study-defined response	Reduction from baseline of ≥ 50% in the HAMD total score	40.0% (12/30)	13.3% (4/30)	*P* < 0.05
	Study-defined remission	Reduction from baseline of ≥ 75% in the HAMD total score	13.3% (4/30)	3.3% (1/30)	*P* > 0.05
(30) (China)	Study-defined response	Reduction from baseline of ≥ 50% in the HAMD total score	46.7% (7/15)	0% (0/15)	*P* < 0.05
	Study-defined remission	Reduction from baseline of ≥ 75% in the HAMD total score	0% (0/15)	0% (0/15)	*P* > 0.05

FEDN = first-episode and drug-naïve; HAMD = Hamilton depression rating scale; NR = not reported; LF-rTMS = low-frequency repetitive transcranial magnetic stimulation.

### Cognitive function

Although 66.7% (2/3) of RCTs investigated the cognitive effects of active LF-rTMS versus sham LF-rTMS (Supplementary Table 1), their data measured by using different measures were not pooled. As shown in Supplementary Table 1, two RCTs consistently found that active LF-rTMS provided a significant improvement in cognitive function over sham LF-rTMS as measured by the Wisconsin Card Sorting Test (WCST) (31) and the cognitive subscale of HAMD (30), respectively.

### Dropout rate and adverse events

As depicted in Supplementary Table 2, none of the included RCTs reported the dropout rate. Only one RCT (30) (33.3%, 1/3) reported adverse events, finding no significant difference regarding dizziness, nausea, or insomnia between the two groups (all *p* > 0.05; Supplementary Table 2).

## Discussion

To the best of our knowledge, this article is the first systematic review of RCTs to investigate the effectiveness and safety of active LF-rTMS versus sham LF-rTMS for children and adolescents (6–25 years) with FEDN MDD. Only three RCTs (29–31) involving 130 subjects with FEDN MDD among children and adolescents were included in this systematic review. The major findings of this systematic review were as follows: (1) active LF-rTMS was more efficacious than sham LF-rTMS in terms of the study-defined response rate and the improvement of cognitive function; and (2) there is a strong indication that LF-rTMS was relatively safe and well tolerated in subjects with FEDN MDD among children and adolescents, although better quality studies are warranted.

In this systematic review, LF-rTMS as a stand-alone treatment appears to be effective for children and adolescents with FEDN MDD, although long-term efficacy was not reported. A recent RCT (*n* = 103) examining the potential therapeutic role and safety of active LF-rTMS versus sham LF-rTMS for adolescents with TRD found that 41.7% responded, and 29.2% met the criteria of remission with active LF-rTMS (40). Numerous RCTs (14) and meta-analyses (2) found that rTMS as an adjunctive therapy is safe and effective in children and adolescents with MDD. Importantly, several recent studies found that LF-rTMS and antidepressants were equally efficacious in reducing depressive symptoms in children and adolescents with MDD (41). Taken together, these findings provide preliminary support for the utility of LF-rTMS in children and adolescents with MDD.

For other noninvasive brain stimulations, such as transcranial direct current stimulation (tDCS) (42–44) and electroconvulsive therapy (45, 46), another objective is to monitor the cognitive effects of rTMS. Consistent with previous meta-analyses focusing on adult patients with MDD (47, 48), this systematic review also found that a therapeutic rTMS course for child and adolescent patients with FEDN MDD may produce modest cognitive enhancing effects. A possible explanation is that cognitive effects were secondary to mood improvement (47). However, the WCST and the HAMD measure used in the included two RCTs (30, 31) do not appear to be suitable for evaluating cognitive performance in MDD. The Assessment of Neuropsychological Status (RBANS) (49) or the MATRICS Consensus Cognitive Battery (MCCB) (50) should be recommended to assess cognitive performance in individuals experiencing MDD in clinical trials. Thus, the cognitive effects of active LF-rTMS compared to sham LF-rTMS should be further examined in FEDN MDD patients among children and adolescents. A recent RCT found that rTMS and tDCS (rTMS-tDCS) than single-tDCS produced greater improvement in neuropsychiatric symptoms (51). As a type of noninvasive cranial electrical stimulation, transcranial alternating current stimulation (tACS) can significantly improve depressive symptoms in adults with FEDN MDD (52). However, there have been no head-to-head studies that compared rTMS either with tACS or tDCS in child and adolescent patients with FEDN MDD.

There are several limitations to this systematic review. First, data were not pooled due to the limited number of studies (3 RCTs) with the heterogeneity of significance between the studies. Second, the sample size (*n* = 130), ranging from 30 to 60, was relatively small. Third, the parameters of LF-rTMS used in the three included studies were varied. For example, the number of total pulses (Table 1) varied from 2,000 to 16,000, which may have resulted in different therapeutic effects and adverse effects. The optimal parameters of LF-rTMS as a stand-alone treatment for FEDN MDD patients among children and adolescents remain unclear. Interestingly, a recent RCT found a significant superiority of Stanford neuromodulation therapy (SNT), a neuroscience-informed accelerated intermittent theta-burst stimulation protocol (90,000 total pulses), in improving depressive symptoms in adults with TRD when compared to sham stimulation (53). Thus, the efficacy and the safety of SNT for patients with MDD among children and adolescents should be examined. Fourth, all 3 RCTs (29–31) included in this study were conducted in China and involved only Chinese children and adolescents. Thus, the findings of the present study could not be generalizable to children and adolescents in other countries. Finally, this systematic review has not been registered.

## Conclusion

These findings preliminarily found that LF-rTMS could benefit children and adolescents with FEDN MDD in a relatively safe manner, although further studies are warranted.

## Data availability statement

The original contributions presented in the study are included in the article/Supplementary material, further inquiries can be directed to the corresponding author.

## Author contributions

X-JL and Z-JQ selected studies and extracted the data. WZ reviewed all the data and helped mediate disagreements, wrote the first draft. All authors contributed to the article and approved the submitted version.

## Funding

This study was funded by National Natural Science Foundation of China (82101609), Scientific Research Project of Guangzhou Bureau of Education (202032762), Science and Technology Program Project of Guangzhou (202102020658), Guangzhou Health Science and Technology Project (20211A011045), Guangzhou Science and Technology Project of Traditional Chinese Medicine and integrated traditional Chinese and Western medicine (20212A011018), China International Medical Exchange Foundation (Z-2018-35-2002), Guangzhou Clinical Characteristic Technology Project (2019TS67), Science and Technology Program Project of Guangzhou (202102020658) and Guangdong Hospital Association (2019ZD06). The funders had no role in study design, data collection and analysis, decision to publish, or preparation of the manuscript.

## Conflict of interest

The authors declare that the research was conducted in the absence of any commercial or financial relationships that could be construed as a potential conflict of interest.

## Publisher’s note

All claims expressed in this article are solely those of the authors and do not necessarily represent those of their affiliated organizations, or those of the publisher, the editors and the reviewers. Any product that may be evaluated in this article, or claim that may be made by its manufacturer, is not guaranteed or endorsed by the publisher.
