# Supplementary material for: Low-frequency repetitive transcranial magnetic stimulation for children and adolescents with first-episode and drug-naïve major depressive disorder: A systematic review
Source: Front Psychiatry. 2023 Feb 8;14:1111754. doi: 10.3389/fpsyt.2023.1111754 (PMC10000294; doi:10.3389/fpsyt.2023.1111754)
Supplement: Supplementary file 1 [file Table_1.doc]

**Appendix S1. Methods**

("Transcranial Magnetic Stimulation"[MeSH] OR Trans-Cranial Magnetic Stimulation OR rtms OR tms) AND ("depression"[MeSH] OR depression OR depressive OR depressed OR melancholia) AND (child OR childhood OR children OR adolescent OR adolescents OR puberty OR pubertal OR juvenile OR teen* OR youth OR preschool OR preschool child OR school age OR high school OR student OR paediatric* OR paediatric* OR minors OR boys OR boy OR girl*) AND (first episode OR early phase OR early-phase OR FEP OR recent onset OR untreated OR unmedicated OR non medicated OR undiagnosed OR first diagnosed OR first diagnosis OR drug-free OR antidepressant-free OR medication-free OR drug-naïve OR antidepressant-naïve OR medication-naïve OR treatment-naïve OR never-medicated).

**Supplemental Table 1. Active versus sham LF-rTMS for adolescent patients with FEDN MDD: cognitive function**

| **Study** | **LF-rTMS Protocol**  **-Intensity (%MT)**  **-Frequency (Hz)**  **-Total pulses** | **Cognitive function** | **Findings** |
| --- | --- | --- | --- |
| Feng et al., 2015 (China) | -70  -0.5  -2000 | None | None |
| Han et al., 2019 (China) | -100  -1  -16000 | WCST | Compared with sham stimulation, LF-rTMS can significantly improve cognitive function in adolescents with FEDN MDD. |
| Zhang et al., 2017 (China) | -80  -1  -6000 | Cognitive subscale of HAMD-24 | Compared with sham stimulation, LF-rTMS can significantly improve cognitive function as measured by the HAMD-24 in adolescents with FEDN MDD. |
| Abbreviations: FEDN=first-episode and drug-naïve; HAMD=Hamilton Rating Scale for Depression; MDD=major depressive disorder; MT=motor threshold; LF-rTMS=low-frequency repetitive transcranial magnetic stimulation; WCST=Wisconsin Card Sorting Test. | | | |

**Supplemental Table 2. Active versus sham LF-rTMS for adolescent patients with FEDN MDD: dropout rate and adverse effects**

| **Study** | **Dropout rate** | **LF-rTMS group (n, %)** | **Sham stimulation group (n, %)** | **Findings** |
| --- | --- | --- | --- | --- |
| Feng et al., 2015 (China) | 0 | 0 | 0 | NS |
| Han et al., 2019 (China) | 0 | 0 | 0 | NS |
| Zhang et al., 2017 (China) | 0 | 0 | 0 | NS |
| **Study** | **Adverse effects** | **LF-rTMS group (n, %)** | **Sham stimulation group (n, %)** | **Findings** |
| Feng et al., 2015 (China) | - | - | - | - |
| Han et al., 2019 (China) | - | - | - | - |
| Zhang et al., 2017 (China) | Dizziness | 1 (6.7) | 1 (6.7) | *p>0.05* |
|  | Nausea | 1 (6.7) | - | *p>0.05* |
|  | Insomnia | - | 1 (6.7) | *p>0.05* |
| Abbreviations: FEDN=first-episode and drug-naïve; LF-rTMS=low-frequency repetitive transcranial magnetic stimulation; MDD=major depressive disorder; NS= not significance. | | | | |
